# Alterations in Resident Immune Cells in Prenatal Trisomy 21 Lungs

**DOI:** 10.3390/cells14231866

**Published:** 2025-11-26

**Authors:** Andrew Frauenpreis, Soumyaroop Bhattacharya, Randa Belgacemi, Pauline Sokolskiy, Gail Deutsch, Nicholas Jendzjowsky, Ian A. Glass, Thomas J. Mariani, Denise Al Alam, Soula Danopoulos

**Affiliations:** 1 Lundquist Institute for Biomedical Innovation at Harbor-UCLA Medical Center, Torrance, CA 90502, USA; frauenpr@usc.edu (A.F.);; 2 Center for Children’s Health Research, University of Rochester Medical Center, Rochester, NY 14642, USA; soumyaroop_bhattacharya@urmc.rochester.edu (S.B.);; 3 Department of Laboratory Medicine and Pathology, Seattle Children’s Research Institute, Seattle, WA 98101, USA; 4 David Geffen School of Medicine at University of California-Los Angeles, Los Angeles, CA 90095, USA; 5 Department of Pediatrics, University of Washington School of Medicine, Seattle, WA 98195, USA

**Keywords:** Down syndrome, B cells, transcriptomics

## Abstract

Respiratory tract infections (RTIs) are amongst the leading causes of hospitalizations in children with Down syndrome (DS). Their elevated susceptibility likely stems from structural differences in the airways and immune system abnormalities. The aim of this study was to characterize immune cells in prenatal Trisomy 21 (T21) lungs, potentially explaining vulnerability to RTIs. Single-cell RNA sequencing was used to profile immune cells in prenatal T21 (*n* = 5) and non-T21 (*n* = 4) prenatal lungs. Spatial phenotypes were assessed via fluorescent in situ hybridization and immunofluorescent staining on prenatal lung tissue sections. Gene expression analysis was also performed on isolated immune cells from lung single-cell suspensions. Several major immune cell populations were identified. A total of 84 DEGs were identified in at least 1 of the 14 different clusters. A significant decrease in the percentage of B cells was observed in T21 lungs (FDR = 0.0037, * *p* < 0.05). Furthermore, qRT-PCR demonstrated B cell markers were significantly decreased in T21, including those associated with B cell maturation (* *p* < 0.05 and ** *p* < 0.01). Several of these markers were also decreased at the protein level (i.e., CD20 and CD38; * *p* < 0.05 and ** *p* < 0.01). Our data demonstrate changes in the T21 pulmonary immune system in utero, primarily within the B cell population, which may contribute to the increased susceptibility to RTIs observed in children with DS.

## 1. Introduction

Trisomy 21 (T21), resulting in Down syndrome (DS), is the most common chromosomal abnormality worldwide. It results from partial or full triplication of chromosome 21 and is associated with multisystem developmental abnormalities. This results in transcriptional heterogeneity [1], which likely contributes to the wide range of dysmorphic features and congenital malformations observed within these individuals, including conditions like congenital heart disease and craniofacial abnormalities. Importantly, increased dosage of chromosome 21 interferon receptor genes biases immune transcriptional programs, providing a mechanistic bridge between genetic and phenotypic variation and the immune dysregulation observed [2]. Consistent with the clinical impact of these immune shifts, the leading causes of hospitalizations for individuals with DS are respiratory tract infections (RTIs), especially within the neonatal and pediatric populations (within the first 3 years of life) [3,4].

Respiratory diseases are the second most common cause of death in children with DS, following cardiac defects [5,6]. Not only are these children more susceptible to both upper and lower RTIs, but the likelihood of recurrence is also increased [4]. Pneumonia is the most common cause of acute hospital admissions in children with DS, accounting for approximately 26% of the number of hospitalizations. Furthermore, the hospitalization rate of Respiratory Syncytial Virus infections in children ≤ 2 years of age is 6–8-fold higher in DS as compared to children without DS [7,8], with the risk for mortality being ~9-fold greater [8]. This increased risk of RTIs in children with DS is likely attributed to both structural and cellular dysregulations, including the immune cells. It has been demonstrated that during mid-gestation, the pulmonary immune cells help pattern airway/alveolar maturation and regulate epithelial–mesenchymal crosstalk [9,10]. Consequently, reduced B cell representation and interferon-conditioned Myeloid/NK compartments can impair antigen handling, cytokine balance, and developmental signaling, mechanisms that shape prenatal lung structure and function and increase susceptibility to postnatal infection.

A significant decrease in the absolute total number of leukocytes, lymphocytes, and monocytes has been reported in the circulating cells of children with DS, whereas a 1.5-fold increase in the proinflammatory monocytes, which are associated with chronic inflammation, has been observed [11,12]. Furthermore, our group and others have demonstrated dysregulation of the complement pathway in DS, which is associated with a proinflammatory phenotype [13]. Interestingly, some studies have demonstrated that whereas T cell lymphocytes may reach levels that are considered “normal” over time, the B cells remain severely decreased [12]. Being that RTIs are more prevalent and severe during the first few years of life, it brings into question whether defects in the T21 immune system are initiated in utero. We recently demonstrated that histopathological defects are observed in prenatal T21 lungs starting as early as 16 weeks of gestation [14]. Therefore, in this study, we aim to understand whether defects in the T21 pulmonary immune cell populations are also observed prenatally. This has been achieved by first defining changes in the regulation of gene expression at the cellular level by performing single-cell RNA sequencing on prenatal T21 lungs presenting with developmental anomalies, assessing genes that are dysregulated, and determining changes in cell localization in situ.

## 2. Materials and Methods

### 2.1. Human Tissue/Ethics

De-identified human prenatal lungs with or without Trisomy 21, between 14.7 and 20.1 weeks of gestation, were obtained from the USC Prenatal Tissue Biobank and the University of Washington Birth Defects Research Laboratory (BDRL) (listed in Table S1). Informed consent was obtained by the tissue banks at the time of donation, and the use of these de-identified, banked specimens for this study was reviewed and designated as “non–human subjects research” by the IRBs at Children’s Hospital Los Angeles (CHLA-15-00342; determination 13 August 2015) and The Lundquist Institute (18CR-32223-01; determination 4 September 2020). The only information provided was gestational age and whether there were any known genetic conditions [chromosomal T21, T18, T13] or gestational complications such as low amniotic fluid. Except for T21, all genetic conditions and complications were excluded from the study. Both male and female tissues were collected and used, with control (non-T21) tissues matching in both gestational age and sex with the T21 samples.

A segment of tissue was fixed as previously described for histological analyses [14]. Another segment of tissue was snap-frozen in RNALater (Invitrogen, Cat: AM7021, Waltham, MA, USA) for gene expression analyses, and a third was used for single-cell suspension preparation [15].

### 2.2. Single-Cell Sequencing

Human prenatal lung tissues were dissociated using the Miltenyi Neural Tissue Dissociation Papain-based Kit (Cat.130-092-628) according to the manufacturer’s instructions alongside additional adaptations. Single-cell capture, library production, sequencing, and cell-type annotation were performed as previously described by us and others [16,17,18,19] and were detailed in our recent paper describing the resident cell sequencing analyses [15]. In brief, it was performed as follows: Single-cell capture and library preparation were performed on the Chromium 10X Genomics system with version 3 chemistry according to the manufacturer’s recommendations. Library quality control was performed on the Agilent Bioanalyzer 2100 using the High Sensitivity DNA Kit (Agilent Technologies, Santa Clara, CA, USA cat. no. 5067-4626). Sequencing was conducted on an Illumina NovaSeq (San Diego, CA, USA), and reads were aligned to the GRCh38 reference genome at a depth of approximately 100,000 reads per cell. All single-cell sequencing data analyses were conducted using Seurat v5.0.1. For generation of the analytical dataset, each sample was independently assessed and adjusted for potential ambient RNA contamination using the DecontX de-contamination algorithm [20]. Subsequently, cells either with fewer than 200 genes detected or with >20% mitochondrial genes were removed. Previous studies have shown that high levels of mitochondrial gene expression are indicative of multiplets [21]. Therefore, using the filtering threshold of >20% mitochondrial genes would exclude putative doublets from the analytical dataset. Additionally, an upper-level feature cutoff of 3× the median number of features was imposed by sample to further account for the exclusion of possible doublets. Highly variable genes were identified using the SCTransform() function of Seurat. Principal component analysis (PCA) was used for dimensionality reduction based on only the highly variable genes. Top contributing principal components (PCs) were identified by Elbowplot() graphs.

Filtered data were normalized, with mitochondrial percentage regressed out, and scaled using the SCTranform() function as implemented in Seurat. Data were integrated using reverse principal component analysis (rPCA) [22], followed by clusterization and visualization using Uniform Manifold Approximation and Projection (UMAP) dimensionality reduction. A cluster resolution of 0.4 was chosen. For single cells, cluster markers were defined through a non-parametric Wilcoxon rank sum test at a significance level of adjusted *p* < 0.05, and corrected for multiple testing using Bonferroni, the default for FindAllMarkers(). For each cell type, differential expression within each cluster was assessed using a non-parametric Wilcoxon rank sum test at a significance level of adjusted *p* < 0.05.

### 2.3. Immune Cell Isolation

Following single-cell suspension preparation, cells were resuspended in FACS buffer [(PBS (95%), FBS (2%), HEPES (2.5%), EDTA (0.4%), and Primocin (0.1%)], quantified, and incubated with CD45 antibody magnetic beads (130-045-801, Miltenyi, Bergisch Gladbach, Germany) for 30 min at 4 °C in the dark. Cells were then washed with FACS buffer and loaded onto MACS LS columns (130-042-401, Miltenyi) at 10^7^ cells per column. Columns were then affixed to a QuadroMACS Separator (130-091-051, Miltenyi) and cells were flushed from the column into a Falcon tube with 5 mL of FACS buffer as recommended by the manufacturer. CD45− and CD45+ fractions were kept separate and were quantified. PTPRC was assessed in both the positive (CD45 +) and negative (all other cell types) isolated fractions, validating the purity of the immune cell fraction (* *p* = 0.0031) (Supplementary Figure S2). Cell viability, quantified by trypan blue immediately before and after magnetic selection, was 82.7 ± 4.6% pre-selection and 87.0 ± 2.6% post-selection across donors. In the magnetically enriched fraction derived from the single-cell suspension, 6.0 ± 1.3% of cells were immune (CD45+).

### 2.4. Quantitative Real-Time PCR Analyses

RNA from CD45+ and CD45- isolated cells were extracted using Easy-Spin™ Total RNA Extraction Kit (Boca Scientific/iNtRON Biotechnology, Cat: 17221, Dedham, MA, USA). cDNA was synthesized using Tetro cDNA synthesis kit (Bioline, Cat: BIO-65043, Taunton, MA, USA). A total of 12.5 ng of cDNA was amplified using specific TaqMan gene expression assays (listed in Table S2; Applied Biosystems, Foster City, CA, USA) and the TaqMan Universal PCR Master Mix II (Applied Biosystems). qRT-PCR products were detected using QuantStudio 3 (Applied Biosystems). Each sample was run in triplicate.

### 2.5. Fluorescent In Situ Hybridization (FISH) and Immunofluorescence (IF) Staining

Human prenatal lung tissues were fixed overnight in 4% PFA at 4 °C, paraffin-embedded, and sectioned. Combinatorial ACD RNAScope FISH and IF staining were performed on lung tissues (T21 and non-T21 age- and sex-matched controls) according to the manufacturer’s instructions and as previously described [13]. Briefly, for FISH staining, fresh 5 µm thick paraffin sections were de-paraffinized in a series of xylene and ethanol. Ready-to-use ACD hydrogen peroxide (ACD Bio, Newark, CA, USA Cat# 322335) was added to the tissue and kept at room temperature (RT) for 10 min. Target retrieval was performed by boiling the sections in 1X RNAscope Target Retrieval Reagent for 15 min at 98 °C on a hot plate (ACD Bio Cat# 322000) and further incubated with ACD Protease plus solution (ACD Bio Cat# 322331) at 40 °C for 25 min. Slides were then incubated, washed, and hybridized with assigned probes at 40 °C for 2 h. Slides were then submerged in 5× SSC buffer (Fisher Scientific, Hampton, NH, USA Cat# BP1325) at RT overnight. The following day, signal amplification and detection were developed using the ready-to-use reagents within the ACD RNAScope Multiplex Fluorescent V2 Assay (ACD Bio Cat# 323100) according to the manufacturer’s recommendations with OPAL Fluorescent Dyes used for labeling. Upon completion of FISH-IF, slides were washed for 3 min with 1×TBST, blocked in 3% bovine serum album/10% Normal Goat Sera/0.1% Triton-X100 in TBS Blocking Solution (1 h at RT), and incubated with primary antibodies at 4 °C overnight. The following day, slides were washed twice for 5 min in 1×TBST, and slides were then incubated with species-appropriate fluorescent conjugated secondary antibodies for 1 h at RT. Slides were then washed twice for 5 min in 1× TBST, counterstained with DAPI (Invitrogen, Waltham, MA, USA Cat# D3571), and mounted with Prolong Diamond Antifade Mounting Media (Invitrogen Cat# P36961). *PAX5* (Cat No. 469981, ACDbio, Newark, CA, USA) and CD19 (Cat No. 402711-C2) probes were used for FISH, and antibodies are detailed in Table S3.

### 2.6. Image Analysis

Images were obtained using a Leica THUNDER (Wetzlar, Germany) inverted microscope. The different combinations of combinatorial FISH/IF were performed on 5–6 different lungs for each condition (T21 and non-T21). Sections were imaged at 40× magnification, with 10 images assessed per sample. Using HALO Image Analysis Platform Version 3.5.3577 (FISH IF or Highplex FL module, Indica Labs, Inc.; Albuquerque, NM, USA), each pair was individually assessed to assure accurate quantification of RNA copy number, intensity, and protein expression.

### 2.7. Statistical Analyses

Statistical analyses were conducted using GraphPad Prism (Version 10) (GraphPad Software Inc., Boston, MA, USA). For immune cell isolates, group normality was assessed with the Shapiro–Wilk test. When data met assumptions for parametric testing, paired Student’s *t*-tests were used to compare isolated-T21 immune cells with sex- and age-matched non-Trisomy controls, with multiple comparisons corrected using Dunnett’s test. When normality assumptions were not met, Wilcoxon matched-pair signed-rank tests were performed, and *p* values were adjusted using Dunn’s test. Statistical significance was defined as *p* ≤ 0.05.

For whole-lung comparisons, due to the limited number of human samples, non-parametric Wilcoxon signed-rank tests were used as appropriate. A paired Student’s *t*-test was used to detect significant differences between non-T21 and T21 age- and sex-matched lungs. The results were considered significant if *p* ≤ 0.05.

## 3. Results

### 3.1. Immune Cell Populations in T21 Prenatal Lungs

**Figure 1:**
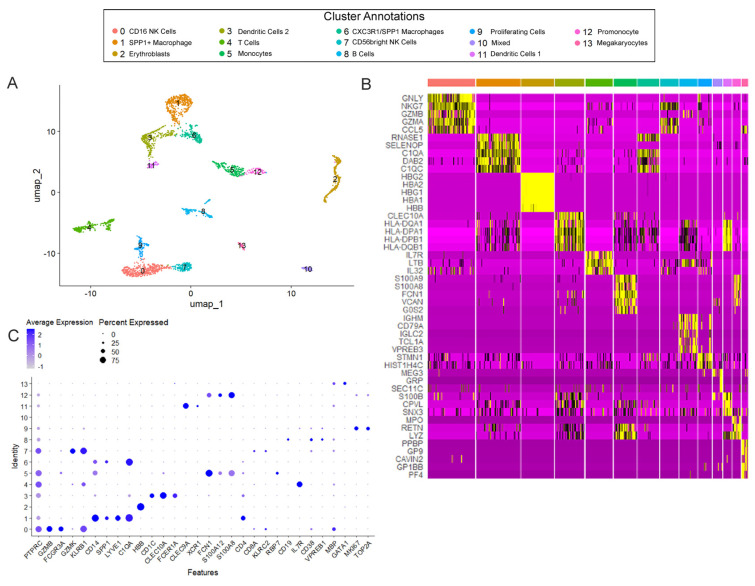
Cellular landscape of pulmonary immune cells in Trisomy 21 (T21) and non-T21 human prenatal lungs. (**A**) Uniform Manifold Approximation and Projection (UMAP) displays the 14 immune cell sub-clusters. Each dot represents a single cell, and individual clusters are colored and annotated based on cell-type associations identified by the ToppFun and human fetal lung cell atlas, using marker genes for each individual cluster. (**B**) The heatmap displays gene expression patterns for marker genes for each cell population cluster, based on differential expression testing. Individual genes are represented in rows, and individual cells are represented in columns. Yellow indicates high relative gene expression, and purple indicates low or no expression. (**C**) The dot plot of canonical immune cell lineage clusters validates that these cells are of immune origin (PTPRC). Expression levels of several specialized markers validate appropriate cell-type annotation, such as SPP1, which is most strongly expressed in cell clusters associated with macrophages, whereas GZMK is solely found in the CD56bright NK cells (Cluster 7). Within these populations, the percentage of cells expressing the gene (using dot size) and the average expression level based on unique molecular identifier (UMI) counts (depth of color) were identified.

Cellular heterogeneity of the immune cells in the developing human prenatal T21 lungs, compared to age-matched controls, was determined via single-cell RNA sequencing. Transcriptomic profiles were generated for the individual human lung cells in tissue obtained from T21 (*n* = 5) and non-T21 (*n* = 4) prenatal lungs. As described in our previous studies, approximately 70% of prenatal T21 lungs in the late pseudoglandular/early canalicular stages of development (16+ weeks of gestation) present with histopathological anomalies [14]. To gain a deeper understanding of the cellular defects in T21 lungs with anatomical abnormalities, all samples were from 17–19 weeks of gestation, with all T21 samples exhibiting anomalies. This manuscript is focused on the immune cells; all other cell types in this dataset were previously analyzed and reported upon separately [15]. Of the total 32,054 cells that were sequenced (non-T21: 16,709; T21: 15,345), 2818 were immune cells (non-T21: 1408; T21: 1410). This accounts for approximately 8–9% of the total prenatal late pseudoglandular/early canalicular lung population, which is consistent with previous studies [9]. Cluster analysis identified the same fourteen immune cell clusters in both the T21 and non-T21 cells (Figure 1A) with no sample level bias (Supplemental Figure S1A). Using the individual cluster markers, multiple immune cell populations were identified, including natural killer (NK) cells, B cells, T cells, macrophages, and dendritic cells (DCs). Cell-type annotations were determined using cluster markers (Figure 1B) and ToppFun/ToppCell Atlas, specifically using the datasets from the Human Fetal Lung in Development Atlas, the Human Embryonic and Fetal Stage Lung Cell Atlas, and The Integrated Human Lung Cell Atlas. Additionally, we assessed the fidelity of our dataset and found strong agreement in the markers of the cell types across two previously published datasets [9,23] (Figure 1C). A complete list of immune cluster marker genes may be found in Supplemental Table S4.

### 3.2. The Developing Human Lung Has Different Immune Cell States

**Figure 2:**
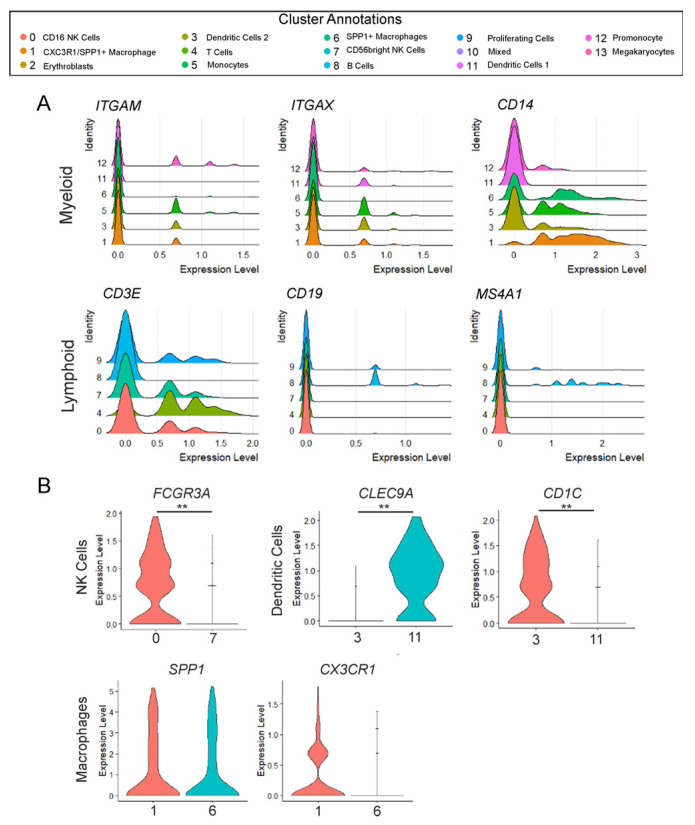
Variations in expression of lineage markers within distinguished clusters of the same immune cell subtypes. (**A**) Ridge plots highlighting the “Myeloid” cell clusters (Clusters 1, 3, 5, 6, 11, and 12) of innate immune cell markers (ITGAM, ITGAX, CD14) portray each cluster as unique based on differing expression levels. Additionally, ridge plots focusing on the “Lymphoid” cell clusters (Clusters 0, 4, 7, 8, and 9) display markers distinct to certain adaptive immune cell types (CD3E, CD19, *MS4A1*), demonstrating that each cluster has a unique transcriptional profile. The *x*-axis represents the normalized expression level whereas the *y*-axis is indicative of the relative proportion of cells expressing the gene. (**B**) Violin plots displaying differences in gene expression between multiple clusters annotated as the same cellular subtypes. FCGR3A is increased in Cluster 0 NK cells compared to Cluster 7 NK cells. CLEC9A is increased in Cluster 11 dendritic cells (DC1), whereas CD1C is increased in Cluster 3 dendritic cells (DC2). SPP1 and CXC3CR1 are increased in Cluster 1 macrophages compared to Cluster 6. ** *p* < 0.0001.

Unlike the non-hematopoietic cell lineages (such as epithelial, mesenchymal, and endothelial), which often share common canonical markers across their subtypes, immune cell types are typically more distinct and uniquely defined. Therefore, we used canonical markers based on class (i.e., Myeloid: ITGAM (CD11b)) and cell type (T: CD3E; B cells: CD19, *MS4A1* (CD20), etc.) to further confirm cell types (Figure 2A). Using ridge plots to assess comparative expression levels of ITGAM (CD11b), ITGAX (CD11c), and CD14, it was confirmed that Clusters 1, 3, 5, 6, 11, and 12 are members of the Myeloid class. However, it is of note that the lack of ITGAM expression in Cluster 11 validates that it is a DC1, as these cells are generally low or negative for this marker. Alternatively, for the Lymphoid lineage cells, there is no single marker that is exclusively and universally expressed. Therefore, using cell-type-specific markers, we validated the presence of T cells (CD3E) and B cells (CD19 and *MS4A1*/CD20) (Figure 2A). However, along with verifying the presence of the different immune cell types, some cell populations appeared to show the presence of different cell subsets: Clusters 0 and 7 being NK cells; Clusters 3 and 11 being dendritic cells; and Clusters 1 and 6 annotating macrophages. The unique identity of each cluster was further supported by analyzing markers exclusive to the specific subclasses. For example, it is known that NK cells are typically divided into two major subsets based on their expression for CD56 and CD16. Assessing FCGR3A expression (which encodes CD16a) via violin plot clearly distinguishes Clusters 0 and 7: Cluster 0 represents the CD56 dim CD16+ cells, known for their high cytotoxic activity, whereas Cluster 7 corresponds to the CD56 bright CD16-negative NK cells, characterized by their cytokine production (Figure 2B, ** *p* < 0.001). These results were further confirmed via co-expression analyses (Supplementary Figure S3). Additionally, it has been well-described that there are two conventional dendritic cell types: DC1 and DC2. Each of these cell types have specialized functions in driving T cell responses. DC1 cells are known to be important for the activation of CD8+ cytotoxic T cells and for promoting Th1 responses [24] whereas DC2s are responsible for the activation of CD4+ T cells and promoting Th2 and Th17 responses [25]. They are distinguished through the expression of different key markers, with DC1 strongly expressing CLEC9A (DNGR-1) and DC2 expressing CD1C (BDCA-1). The difference in expression of these two markers between Clusters 3 and 11 clearly demonstrates that Cluster 3 is DC2 whereas Cluster 11 represents the DC1 (Figure 2B, ** *p* < 0.001), which was further confirmed via co-expression analyses (Supplementary Figure S3). Finally, it was also noted that two sets of macrophages were present, with Cluster 1 being highly expressive for CX3CR1 (Figure 2B) compared to Cluster 6, which is thought to contain the non-classical anti-inflammatory macrophages found in tissue. Altogether, the thorough assessment of the different clusters demonstrates how complicated the pulmonary immune system is, even during in utero development.

### 3.3. Significantly Fewer B Cells in Prenatal T21 Lungs

**Figure 3:**
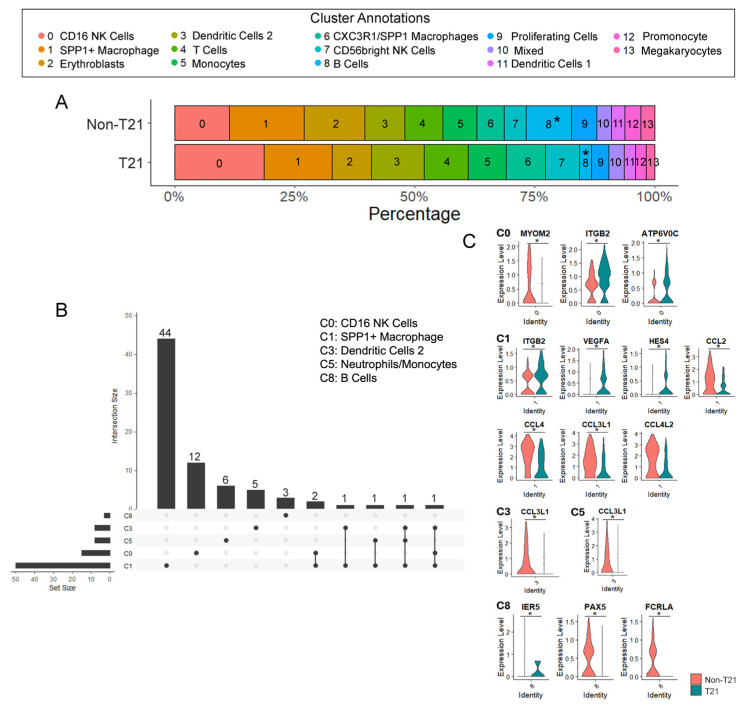
Differential expression analyses indicate immune system dysregulation initiates in utero in prenatal T21 lungs. (**A**) Proportional differences in immune sub-clusters between non-T21 and T21 cohorts show T21 lungs have a significant reduction in Cluster 8 B Cells (FDR = 0.0037; * *p* < 0.05). (**B**) A total of 84 genes were significantly differentially expressed in T21 in one or more cell clusters. Shown here is an upset plot indicating the number and intersection of differentially expressed genes (DEGs) identified within each cluster of immune cells. Horizontal bars on the left represent the total number of DEGs within each individual cluster. Vertical bars indicate the size of each intersection, i.e., the number of genes shared among specific clusters, with connected dots below each bar showing the corresponding combination of spot types on the *x*-axis. (**C**) Violin plots demonstrating select statistically significant DEGs within different clusters between non-T21 and T21 cohorts. * *p* < 0.05.

As mentioned, the proportionality of total immune cells between the T21 and non-T21 prenatal lungs was comparable, ranging between ~8 and 9% in both conditions [9]. However, proportional changes in the distribution of the specific immune cell types needed to be further assessed, as several previous studies have already demonstrated a significant decrease in B cells [26], altered differentiation in T cells [27,28], and age-related changes in number and cell activity of NK cells [11,29] within the peripheral blood of children and adults with DS. Although there seemed to be a trend toward a higher proportion of NK cells in T21 when considering Clusters 0 and 7 together, the only cell population in which a statistically significant adjusted *p* value was observed (FDR = 0.0037, *p* = 0.0003) was the B cells (Cluster 8), demonstrating a decrease in prenatal T21 lungs, averaging around 2.47% of total T21 immune cells compared to non-T21, which averaged around 9.38% of total non-T21 immune cells (Figure 3A).

In addition to understanding what proportional differences were observed between the T21 lung immune cells and non-T21, it was also necessary to understand how their transcriptomic profiles differed. We identified a total of 84 differentially expressed genes (DEGs) in at least 1 of the 14 different clusters (FDR < 0.05), with the SPP1+ macrophages (Cluster 1) presenting with greatest number of DEGs at 50 (Figure 3B). This may be noted through the upset plot (Figure 3B) indicating the number and intersection of DEGs identified within each cluster of immune cells. Interestingly, despite a marked proportional decrease in B cells in the prenatal T21 lungs, only three DEGs were identified in this cluster (Figure 3C: IER5, *PAX5*, and FCRLA), likely due to limited statistical power from the small number of cells in the dataset (Supplemental Figure S1C T21: 37; non-T21: 129). Furthermore, we observed downregulation of multiple chemokines (CCL2, CCL3L1, CCL4, CCL4L2) involved in immune cell recruitment within the macrophages (Cluster 1), as well as genes that have been shown to be important in immune cell function and development (*PAX5*, FCRLA) (Figure 3C). A complete list of differentially expressed genes for each immune cluster is presented in Supplemental Table S5.

### 3.4. B Cell Development and Maturation Is Hindered in Prenatal T21 Lungs

Given previous findings showing a reduction in B cells in children with DS and the decrease observed in this single-cell dataset in prenatal T21 lungs, we aimed to further investigate and validate whether B cells are indeed reduced or localized differently during development and to better understand their role through gene expression analysis. Using a magnetic bead/column separation technique, we isolated the immune cells (CD45+) from T21, and sex/age matched non-T21 control prenatal lung single-cell suspensions that were different from the samples processed for the scRNAseq dataset. We were then able to perform additional gene expression analyses on these isolates. PTPRC was assessed in both the positive (CD45+) and negative (all other cell types) isolated fractions, validating the purity of the immune cell fraction (* *p* = 0.0031) (Supplementary Figure S2).

**Figure 4:**
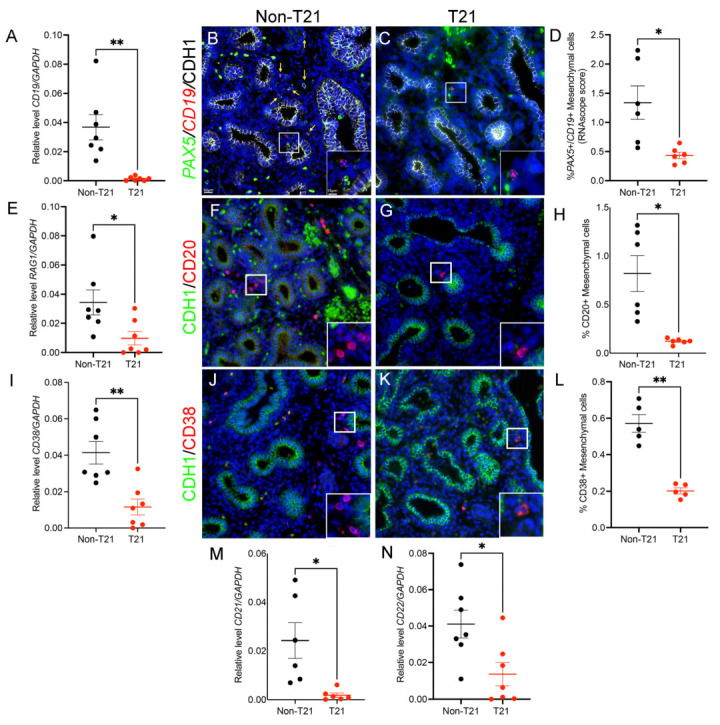
Altered expression of B cell development and maturation markers in prenatal T21 lungs. (**A**) qRT-PCR analysis of CD19 in immune cells isolated from T21 and non-T21 prenatal lungs, showing significantly decreased expression in T21 samples (*n* = 7). (**B**,**C**) Representative FISH images for CD19 (red) and *PAX5* (green) combined with CDH1 (white) IF staining in non-T21 (**B**) (*n* = 6) and T21 (**C**) (*n* = 6) prenatal T21 lung sections. (**D**) Quantification of double-positive *PAX5*/CD19 cells using RNAscope scoring, confirming a significant reduction in B cells in T21 lungs compared to non-T21 controls (*n* = 6). (**E**) qRT-PCR analysis of RAG1 gene expression demonstrates significantly reduced expression of T21 lung immune cells (*n* = 7). (**F**,**G**) Representative IF images for CD20 (red)/CDH1 (green) in non-T21 (**F**) and T21 (**G**) prenatal lungs, and associated quantification (**H**), (*n* = 6) demonstrating a substantial decrease in CD20-positive cells in T21 lungs. (**I**) qRT-PCR analysis shows decreased CD38 expression in immune cells isolated from T21 lungs (*n* = 7). (**J**,**K**) Representative IF images of CD38 (red)/CDH1 (green) in non-T21 (**J**) and T21 (**K**) prenatal lungs. (**L**) Quantification of CD38-positive cells confirming a significant reduction in T21 lungs (*n* = 5). Expression analysis of mature B cell markers CD21 (**M**) (*n* = 6) and CD22 (**N**) (*n* = 7), demonstrating significantly lower expression in T21 lungs compared to controls. Results are shown as individual data points and mean ± SEM; * *p* < 0.05; ** *p* < 0.01. Scale bars are indicated by 50 µm and 15 µm for large and small inserts, respectively. Panels (**A**,**D**,**H**,**I**,**L**–**N**) were analyzed using paired two-tailed Student’s *t*-tests with Dunnett’s correction for multiple comparisons; panel (**E**) (RAG1) was analyzed using a Wilcoxon matched-pairs signed-rank test with Dunn’s correction.

To better assess B cell populations in T21 prenatal lungs, we evaluated their expression and localization using a panel of B cell markers (Figure 4). qRT-PCR for the general B cell marker CD19, which is part of the B cell co-receptor complex, showed a significant decrease in the expression of immune cells isolated from T21 lungs as compared to non-T21 (0.0013 ± 0.0005 vs. 0.0367 ± 0.0086; *n* = 7; *p* = 0.007; Figure 4A). These results were confirmed at the whole-tissue level by FISH for CD19 and *PAX5*, a transcription factor essential for B cell development and function, in non-T21 (Figure 4B) and T21 (Figure 4C) prenatal lung sections. Quantification using RNAscope scoring demonstrated a significant reduction in the percentage of double-positive *PAX5*/CD19 cells in T21 lungs (0.43 ± 0.05 vs. 1.33± 0.28; *n* = 6; *p* = 0.026; Figure 4D). We next investigated RAG1 expression (Figure 4E), a marker of early B cell development that is involved in the V(D)J recombination, which is the component that allows for the structuring of a unique B cell receptor. Similarly to the aforementioned markers, RAG1 was significantly reduced in T21 lung immune cells compared to non-T21 samples (0.009 ± 0.0004 vs. 0.03 ± 0.008; *n* = 7; *p* = 0.026). Finally, in association with early B cell markers, we also evaluated the localization of CD20+ cells in prenatal lung tissues, which are first expressed in the late pre-B cell stage and are a calcium channel regulator [30,31]. IF staining for CD20 (Figure 4F,G) revealed a drastic reduction in the percentage of CD20-positive cells in T21 lung tissues as compared to non-T21 (0.12 ± 0.01 vs. 0.81 ± 0.18; *n* = 6; *p* = 0.01; Figure 4H).

In addition to determining whether the development of B cells was altered in the prenatal T21 lungs, we sought to determine whether maturation of these cells was dysregulated. CD38, which is upregulated during B cell activation, was significantly decreased in the isolated T21 lung immune cells (0.01 ± 0.004 vs. 0.04 ± 0.006; *n* = 7; *p* = 0.001; Figure 4I), which was also observed by IF staining in whole-lung tissue (Figure 4J,K) (0.20 ± 0.01 vs. 0.57 ± 0.04; *n* = 5; *p* = 0.004; Figure 4L). Furthermore, the analysis of CD21 (Figure 4M), a marker known to help maintain memory B cells, and CD22 (Figure 4N), known for its role in maintaining immune tolerance and reducing the risk of autoimmunity, both showed a significant decrease in T21 lungs compared to controls (respectively, 0.001 ± 0.0009 vs. 0.02 ± 0.007; *n* = 6; *p* = 0.024 and 0.01 ± 0.006 vs. 0.04 ± 0.007; *n* = 7; *p* = 013). Altogether, these data reveal a global reduction in B cell populations during development in T21 prenatal lungs, suggesting impaired B cell development, maturation, and local immune establishment in this context.

## 4. Discussion

It has been well-established that the immune system in individuals with DS is dysregulated and functionally altered, yet no studies have specifically investigated the immune system within the developing T21 lung [32,33]. This is especially important given that the lung’s immune system is uniquely adapted to its constant exposure to the external environment, requiring it to remain responsive to inhaled pathogens. Furthermore, the pulmonary immune system has several cell types that solely reside in the lung, such as lung-resident basophils and alveolar macrophages [34]. Although the prenatal lung is not exposed to these external pathogens, studies have shown that the pulmonary immune system is established in utero, with the majority of cells being tissue-resident rather than derived from circulating blood within the lung vasculature [9,34]. This has been confirmed in our current study, validating the presence of several adaptive and innate immune cell types (Figure 1). Therefore, given what is known regarding the dysregulated immune system in children and adults with DS, it is critical to understand when these defects initiate within the prenatal T21 lung.

The adaptive immune system of individuals with DS has been classified as being impaired or underdeveloped. Particularly in children with DS, multiple studies have reported a significant reduction in the absolute numbers of T and B cells [12]. This is of interest given that even within our limited single-cell dataset, we were still able to observe a statistically significant decrease in B cell numbers within prenatal T21 lungs (Figure 3A). Previous research has suggested that increased lymphocyte apoptosis may contribute to the lymphopenia observed in DS, particularly within the B cell population [35,36]. However, we did not assess apoptosis in our study, so this mechanism remains speculative in the context of our findings. Furthermore, our FISH/IF and gene expression analyses validated that not only do prenatal T21 lungs present with fewer B cells, but they are less mature, indicated by the decreased expression of markers such as CD38, CD21, and CD22 (Figure 4). This likely signifies the point when memory B cell development becomes impaired in these individuals [37,38]; cells that are essential for long-term immunity and effective vaccine responses are notably deficient within this population. It has been demonstrated that approximately 62% of individuals with DS present with abnormal pneumococcal titers post pneumonia vaccination [39] and that only 27% of children with DS reach the defined protective level in response to the influenza A/H1N1 vaccination [40]. Therefore, the body’s inability to develop antigen-specific immunity via vaccine response, alongside the additional complications noted in the adaptive immune system, are likely the main contributors to increased susceptibility to respiratory viral infections observed in children with DS. This correlates with the significantly decreased *PAX5* (*p*-adjusted: 0.0017) expression observed in the B cell cluster of our scRNAseq dataset. Furthermore, the aberrant prenatal immune system likely primes for several of the autoimmune conditions that are prevalent in children with DS, including diseases such as type I diabetes, alopecia areata, and celiac disease. It has been demonstrated that by 2 years of age, 17% of children with DS are diagnosed with type I diabetes, which is drastically increased compared to the 0.4% observed in general population [41].

Recent deep immune phenotyping studies in individuals with DS have highlighted not only widespread immune dysregulation but also substantial interindividual variability in immune phenotypes, including lymphocyte subset composition, cytokine profiles, and immune-mediated comorbidities across the lifespan [1,42,43,44]. This emerging heterogeneity contrasts with the relatively homogeneous reduction in lung B cells that we observe across prenatal T21 samples. One plausible explanation is that our study captures a restricted developmental window in utero, during which T21-driven perturbations in lung B cell development may be more stereotyped, whereas postnatal environmental exposures, infections, and additional genetic and epigenetic modifiers progressively diversify immune trajectories. In this framework, a shared early lesion in lung B cell development could provide a common substrate upon which heterogeneous systemic immune phenotypes later emerge in children and adults with DS.

Mechanistically, we can hypothesize that overdosage of the four interferon receptor genes on HSA21 (IFNAR1, IFNAR2, IFNGR2, IL10RB) creates a chronic, low-grade, type I/II interferonopathy that perturbs early B cell development in the lung, as supported by genetic and functional studies in blood and mouse models [45,46,47,48]. Although our current dataset is underpowered to formally test interferon or JAK-STAT transcriptional programs within individual clusters, our findings are consistent with the idea that B cell developmental trajectories in T21 lungs are already being shaped in an interferon-rich environment, in line with the broader concept of DS as an interferonopathy.

Another typical feature of DS is mitochondrial and metabolic dysfunction across multiple tissues, including immune cells, where altered oxidative phosphorylation, redox balance, and mitochondrial quality control have been reported by us and others [49,50,51,52]. This is particularly relevant for B cells, which depend heavily on mitochondrial metabolism during activation, class-switch recombination, and memory formation. In healthy donors, B cells increase oxidative phosphorylation and undergo marked mitochondrial remodeling upon activation, and mitochondrial quality control is crucial for the long-term survival of memory B cells [53,54,55]. Future analyses of our single-cell dataset focusing on transcriptional surrogates of mitochondrial function (e.g., electron transport chain components, TCA cycle enzymes, and mitophagy regulators) in the B cell clusters could therefore help to determine whether early mitochondrial dysregulation contributes to the altered B cell distribution and maturation that we observe in prenatal T21 lungs.

In addition to presenting with compromised adaptive immunity, individuals with DS also exhibit dysregulation of the innate immune system. Contrary to the trend observed in other lymphocytes, studies have demonstrated an increased number of NK cells in the blood of children with DS [11,56], possibly reflecting precocious immune development or compensatory response to reduced adaptive immunity. However, functional analyses reveal that NK cell activity is markedly diminished, further enhancing their susceptibility to things such as respiratory viral infections [29,56]. This progression of development appears to be initiated in utero, as we noted a similar increasing trend in our two NK cell populations (Clusters 0 and 7) within the T21 lungs; however, significance was not reached provided the stringent FDR adjustment parameters were used. Notably, we observed decreased expression in genes such as *KLRC1* (*p*-adjusted: 0.0004), an inhibitory receptor of NK cells whose loss of regulation may lead to impaired NK-mediated killing [57], and *HLA-B* (*p*-adjusted: 0.003), an MHC Class I molecule that can influence NK cell education [58]. Taken together with adult DS cohorts that show broad NK and T cell remodeling and chronic interferon pathway activation [48,59], our data support a model in which innate and adaptive abnormalities begin in the prenatal period and then diversify during postnatal life under environmental and infectious pressures.

In this study, we demonstrate that pulmonary immune defects initiate in utero for individuals with DS, potentially priming their heightened susceptibility to respiratory viral infections observed within the neonatal and pediatric populations. While we acknowledge limitations in our single-cell sequencing dataset, particularly the low capture of immune cells, provided that the prenatal lung solely comprises 8% immune cells (our recovery rate), the pronounced differences in the B cell population underscore the significance of our findings. Validation across additional immune cell isolates and tissue sections further confirms that B cell development and maturation are compromised early in DS. Additionally, we acknowledge that given that limited number of immune cells, the number of significantly different genes within individual clusters is in fact too small and as such lacks power to derive valid results from any kind of functional assessment or pathway analysis. Therefore, sequencing a larger cohort would be beneficial in establishing alterations in immune cell functionality and molecular dysregulation. In addition, while IF stainings confirm a reduced B cell population in T21 lungs, proteomic validation would further strengthen and consolidate these observations. Although further investigation is needed to elucidate the roles of other developing pulmonary immune cell types, our current study provides critical insight into the early immune cell landscape of T21 lungs and helps us better determine why children with DS are more susceptible to viral infections and autoimmunity early in life.

## Data Availability

The datasets generated during and/or analyzed during the current study are available in the NCBI SRA/GEO repository, Accession Number: GSE272470. Additionally, code used in analyses may be found at https://github.com/sdanopoulos/git_Danopoulos_T21_lung_sc (accessed on 28 May 2024).

## Supplementary Materials

The following supporting information can be downloaded at https://www.mdpi.com/article/10.3390/cells14231866/s1: Figure S1: Prenatal T21 immune cell quality control, Figure S2: PTPRC Expression confirms purity of CD45+ Immune Cell Fraction, Figure S3: Gene co-expression to validate cell type identification; Table S1: Tissue Identification, Table S2: TaqMan Probes, Table S3: Antibody List, Table S4: Immune Cluster Marker Genes, Table S5: Immune Cluster DEGs.
