# Supplementary material for: Alterations in Resident Immune Cells in Prenatal Trisomy 21 Lungs
Source: Cells. 2025 Nov 26;14(23):1866. doi: 10.3390/cells14231866 (PMC12691487; doi:10.3390/cells14231866)
Supplement: Supplementary file 1 [file cells-14-01866-s001.zip › Supplementary Tables S1-S3 and Figures S1-S3.pdf]

**Supplementary Tables & Figures:**

**Table S1: Tissue Identification**

| Sample Type | Identification | Gestational Age (Wks) | Sex | Application |
|---|---|---|---|---|
| Non-T21 | H29488 | 20.1 | F | Immune cell isolation, IF, FISH |
| T21 | H28811 | 19.6 | F | Immune cell isolation, IF, FISH |
| Non-T21 | H29008 | 20.1 | F | Immune cell isolation IF, FISH |
| T21 | H29493 | 19.3 | F | Immune cell isolation IF, FISH |
| Non-T21 | H29411 | 14.5 | M | Immune cell isolation |
| T21 | H29746 | 14.5 | M | Immune cell isolation |
| Non-T21 | H29345 | 19.1 | M | Immune cell isolation IF, FISH |
| T21 | H29370 | 19.1 | M | Immune cell isolation IF, FISH |
| Non-T21 | H29427 | 16 | F | Immune cell isolation IF, FISH |
| T21 | H29424 | 15.5 | F | Immune cell isolation IF, FISH |
| Non-T21 | H29205 | 16.5 | M | Immune cell isolation |
| T21 | H29910 | 17.3 | M | Immune cell isolation |
| Non-T21 | H28758 | 17.4 | M | Immune cell isolation IF, FISH |
| T21 | H28790 | 18.1 | M | Immune cell isolation IF, FISH |
| Non-T21 | 359 | 19.5 | F | Immune cell isolation IF, FISH |
| T21 | H28433 | 19.4 | F | Immune cell isolation IF, FISH |

**Table S2: TaqMan probes**

| Target gene | Probe ID |
| --- | --- |
| *GAPDH* | HS02786624 |
| *PTPRC* | Hs04189704 |
| *CD19* | Hs01047413 |
| *CD38* | Hs01120071 |
| *CD22* | Hs00998488 |
| *RAG1* | Hs01920694 |
| *SPN* | Hs01872322 |
| *CR2* | Hs00153398 |

**Table S3: Antibody list**

| Antibody | Company | RRID | Host species | Dilution |
| --- | --- | --- | --- | --- |
| CDH1<br>E-Cadherin (24E10) | Cell signaling technology, Danvers, MA, USA | RRID:AB_2291471 | Rabbit | 1/200 |
| CD20 | Proteintech, Rosemont, IL, USA | RRID, AB_2881391 | Mouse | 1/100 |
| CD38 | Proteintech | RRID, AB_716994 | Mouse | 1/100 |

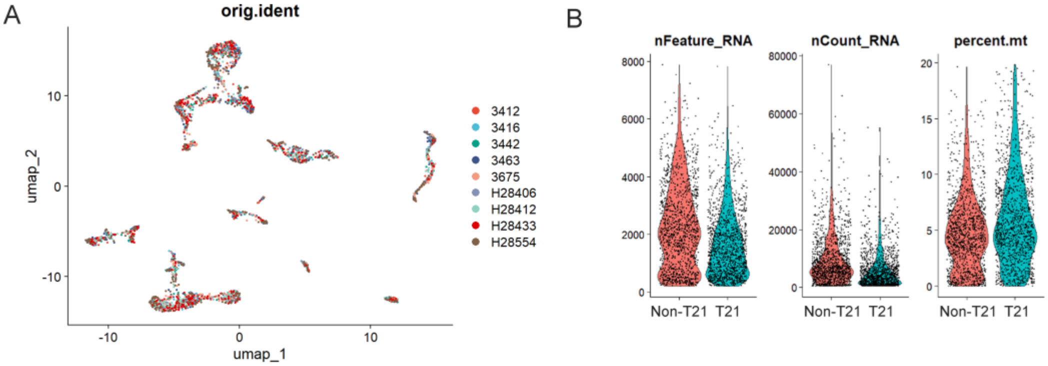


C

| Cluster | Non-T21 | T21 | Total |
|---|---|---|---|
| 0 | 171 | 260 | 431 |
| 1 | 207 | 198 | 405 |
| 2 | 183 | 112 | 295 |
| 3 | 113 | 159 | 272 |
| 4 | 121 | 131 | 252 |
| 5 | 99 | 110 | 209 |
| 6 | 79 | 118 | 197 |
| 7 | 68 | 99 | 167 |
| 8 | 129 | 37 | 166 |
| 9 | 75 | 49 | 124 |
| 10 | 42 | 44 | 86 |
| 11 | 41 | 37 | 78 |
| 12 | 45 | 31 | 76 |
| 13 | 35 | 25 | 60 |
| Total | 1408 | 1410 | 2818 |

**Figure S1. Prenatal T21 immune cell quality control** A) Distribution of epithelial cells by sample in UMAP shows no sample-level bias. B) Quality control panels for genes expressed, UMI counts, and mitochondrial gene proportions within each immune sub-cluster. C) Table shows cell distribution by condition (Non-T21 vs T21) for each sub-cluster.

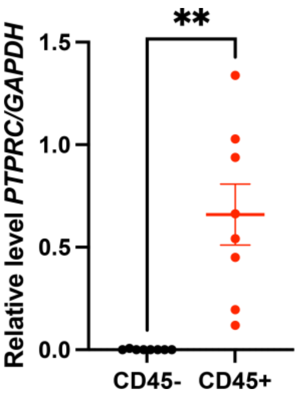


**Figure S2. PTPRC Expression Confirms Purity of $CD45^+$ Immune Cell Fraction** RT-qPCR analysis of PTPRC expression in $CD45^+$ immune cells and $CD45^-$ cells isolated from T21 and non-T21 prenatal lungs. PTPRC transcripts were detected only in the $CD45^+$ fraction, confirming the specificity and efficiency of the $CD45^+$ isolation. Results are shown as individual data points and mean ± SEM; $**p < 0.01$.

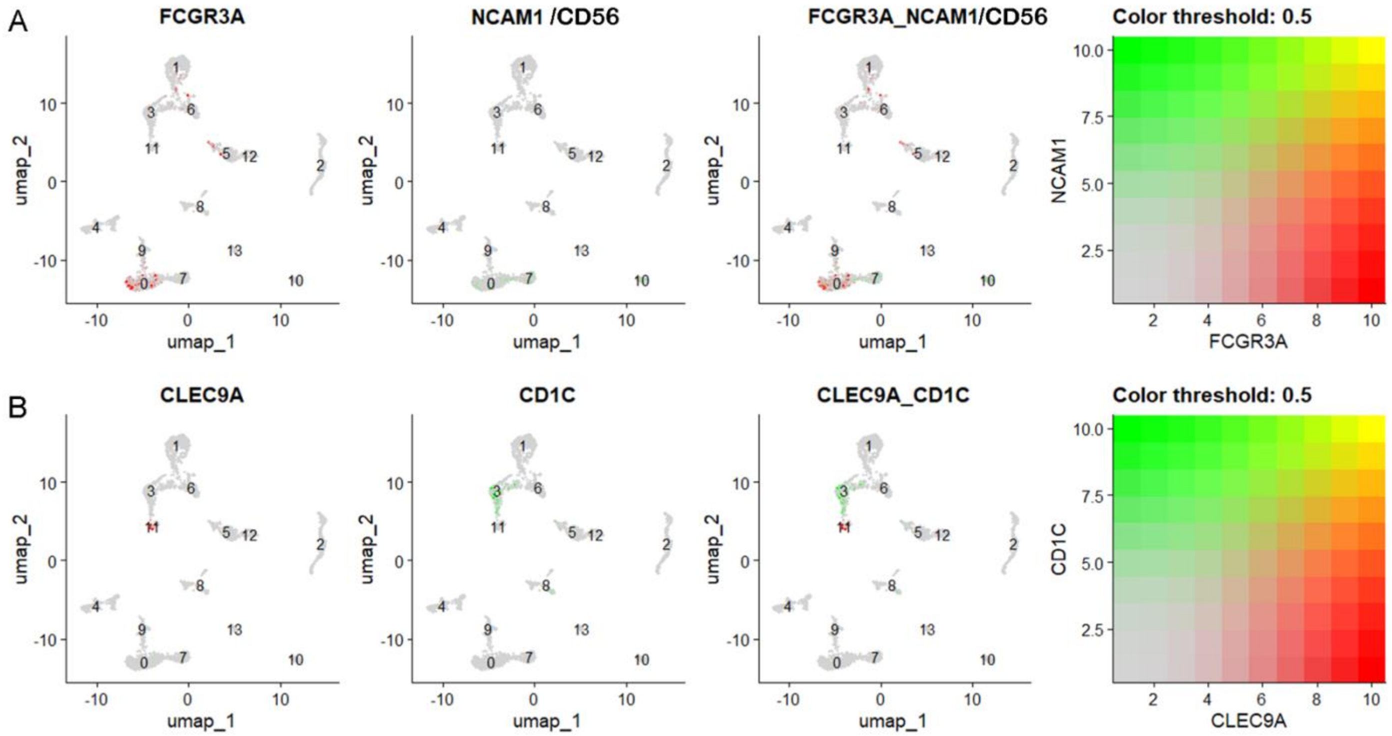


**Figure S3. Gene co-expression to validate cell type identification.** (A) Immune marker co-expression of FCGR3A (Red) and NCAM1/CD56 (Green) represented by UMAP. Each dot represents a single cell. Cells demonstrating FCGR3A/NCAM1 co-expression would be represented in yellow. However, we did not observe any cells co-expressing both markers. (B) Immune marker co-expression of CLEC9A (Red) and CD1C (Green) represented by UMAP. Each dot represents a single cell. Cells demonstrating CLEC9A/CD1C co-expression would be represented in yellow. However, we did not observe any cells co-expressing both markers.
